# Postdigital. Interaktiv. Partizipativ. Mit der PIP-Formel in die Post-Corona-Zeit

**DOI:** 10.1365/s40702-021-00737-9

**Published:** 2021-05-27

**Authors:** Anna M. Lux, Felicitas Macgilchrist

**Affiliations:** 1 Technische Universität Braunschweig, Braunschweig, Deutschland; 2 Georg Eckert Institut – Leibniz-Institut für internationale Schulbuchforschung (GEI), Braunschweig, Deutschland; 3 Institut für Erziehungswissenschaft, Georg-August-Universität Göttingen, Göttingen, Deutschland

**Keywords:** Digitalität, Postdigitalität, Partizipation, Communities, Kollaboration, Digitality, Postdigital, Participation, Communities, Collaboration

## Abstract

Was in der Pandemie deutlich wird, zeichnete sich bereits ab: Das Verständnis von Zusammenarbeit hat sich verändert. Die Mitwirkung in verschiedenen Teams, Netzwerken und Communities ist ebenso selbstverständlich geworden, wie das überregionale und internationale Agieren. Wo Jeder mit Jedem vernetzt ist, stellt nicht die Digitalisierung die Herausforderung dar, sondern die Gestaltung der Prozesse im Miteinander. Das Zusammenspiel von Mensch, Technik und Raum im postdigitalen Zeitalter erfordert Methoden und Arbeitsweisen, die Kreativität, Meinungsvielfalt und Beteiligung fördern. Abgeleitet aus einem Praxisbeispiel möchten wir zur Gestaltung zeitgemäßer Formate der Zusammenarbeit eine Basis vorschlagen: die Anwendung der PIP-Formel im Zusammenspiel mit den Liberating Structures.

## Abstract (en)

The SARS-CoV‑2 pandemic has significant transformed society, but one issue was already visible: The idea of collaboration has changed. We now take it for granted that we will work together in teams, networks and communities, often internationally. When everyone is connected, then the prime challenge is not which new technology to introduce, but how to shape our collaborative practices. The postdigital world, in which humans, technology and space are interwoven, requires methods, tools and practices that support creativity, participation and multiple voices. Taking an in-depth look at one example, this paper identifies a future-ready mode of designing collaborative work: the PIP (postdigital, interactive, participatory) pathway, together with liberating structures.

## Digitalisierung ist nicht mehr die Herausforderung

Der Kurs für die Zusammenarbeit des 21. Jahrhunderts ist gesetzt auf ein Miteinander in Netzwerken und Communities. Die steigende Komplexität globaler Probleme erfordert auf der Suche nach Lösungen eine Befreiung aus disziplinären Strukturen (Ledford 2015). Technische und soziale Innovationen bedingen eine gemeinsame Kreativität, die sich nicht ausbilden kann innerhalb fester Kategorien, strikter Hierarchien und starrer Ordnungen (Lattemann et al. 2020). Interdisziplinarität und Kooperation bilden den Nährboden für Innovations- und Transformationsprozesse in Wirtschaft und Wissenschaft. So werden z. B. interdisziplinäre Teams aus vorher strikt getrennten Abteilungen zur strategischen Unternehmensentwicklung eingesetzt (Bartz und Schmutzer 2015), Innovationssprünge von Produktionsprozessen über die Mitwirkung in Technologieclustern forciert (Klocke 2002) und regionale Partnerschaften an und mit Hochschulen über Netzwerke, Forschungszentren, Reallabore, Hubs oder virtuelle Campi aufgebaut (vgl. Lux und Robra-Bissantz 2020). Dieses neue Miteinander ist ein international wahrnehmbarer Trend (Ledford 2015).

Doch wie werden die Ziele der Zusammenarbeit konkret umgesetzt, vor allem vor dem Hintergrund der SARS-CoV‑2 Pandemie? Bei näherer Betrachtung der Digitalisierung von Arbeitstreffen, d. h. deren Übersetzung in Videokonferenzen, wird sichtbar, dass die eingesetzten Methoden und Kommunikationsformen zu oft dazu führen, dass sich nur Expert*innen und Extrovertierte aktiv beteiligen. Diese Arbeitsweise begrenzt die kreative Zusammenarbeit, viele wertvolle Ideen gehen verloren.

Wird die Zusammenarbeit nicht an der „Digitalisierung“, sondern an der „postdigitalen“ Arbeitsweise ausgerichtet, rückt die Verbindung zwischen Kommunikationsformen, Technologie, dem sozialen Miteinander, produktivem Austausch und einer Beteiligung *aller* Teilnehmenden in den Vordergrund. Mit dem Begriff „postdigital“ wird auf gegenwärtige Gesellschaften verwiesen, in denen es nicht mehr möglich ist, zwischen online und offline bzw. *virtual life* und *real life* zu unterscheiden (Jandrić et al. 2018; Taffel 2016). Das Digitale ist stets auch materiell (ob in Form eines schwachen W‑LANs, ausgelasteter Server oder der Kinder im Homeschooling, die im Bildschirm auftauchen). Auch wenn in vielen Bereichen neue digitale Technologien eingeführt werden, so kann die bloße Digitalisierung an sich nicht mehr als innovativer Prozess gelten. Wir sind alle schon – so die grundlegende Annahme – mittels digitaler Technologien vernetzt; das Smartphone ist stets dabei. Selbst Personen ohne Smartphone oder soziale Medien sind eingebunden: Sobald sie Dienste nutzen, wie z. B. ein Bankkonto, fließen Details ihres Lebens in global vernetzte Datenflüssen ein (Baker et al. 2013). Hybride analog-digitale Technologien und Praktiken prägen unser Wirtschafts‑, Wissenschafts- und Alltagsleben. Disruption ist erwartbar geworden. Nun geht es darum, innovative Formen der Zusammenarbeit zu entwickeln und zu erproben, die uns (postdigital) zusammenbringen.

## Auf dem Weg zur Postdigitalität

### Von neuen Arbeitswelten und alten Methoden

Die Pandemie bestärkt die Bedeutung bereits identifizierter Grundpfeiler neuer Arbeitswelten: offene Strukturen und eine hohe Flexibilität für das Zusammenspiel von Mensch, Technik und Raum. Das geänderte Verständnis von Arbeit erfordert eine Auflösung starrer Hierarchien, die Umsetzung neuer Arbeitszeitmodelle und den Aufbau von Arbeitsumgebungen, die über geeignete Methoden Austausch- und Lernprozesse effektiv fördern (Keller 2017). Neue Denkansätze für Projekt‑, Innovations‑, Portfolio- und Entwicklungsmethoden, haben in den letzten Jahren bereits ein großes Repertoire an Innovationsmethoden geschaffen (Zapfl 2018). Bekannte Beispiele im Bereich der agilen (Projekt‑) Organisation sind Scrum, OKR oder Kanbans, im Bereich der kreativen Techniken zur Lösung komplexer Probleme insbesondere das Design Thinking. Einige dieser multidisziplinären Ansätze sind auch dort für die Arbeits- und Organisationsentwicklung von Interesse, wo bisherige Modelle an ihre Grenzen stoßen.

Um die Zusammenarbeit in einer großen Gruppe unterschiedlicher Disziplinen zum Erfolg zu führen, ist vor allem die Wahl der Kommunikationsformen entscheidend (McCandless und Lipmanowicz 2014). Ziel sollte es dabei sein, über die informative Ebene hinaus zu gelangen und Interaktionen zu ermöglichen und zu unterstützen. Es gilt, *alle* Gruppenmitglieder in Entscheidungs- und Gestaltungsprozesse aktiv einzubeziehen. Konventionelle Kommunikations-Routine, wie sie im heutigen Arbeitsalltag präsent ist, eignet sich hierzu nur bedingt. Die am häufigsten verwendeten Methoden führen zu Ausschluss und Frustration, da sie entweder zu hemmend oder zu unstrukturiert sind. So hindern Präsentationen, Statusberichte oder geführte Diskussionen überwiegend das kreative Engagement der Beteiligten, während offene Diskussionen oder Brainstorming-Runden Introvertierte benachteiligen (Lipmanowicz und McCandless 2013).

### Befreiung aus Mustern vergangener Dekaden: Liberating Structures

Um Zusammenarbeit auf eine neue Ebene zu führen, wurde 2013 eine Sammlung von Moderationsformaten und Methoden zur Förderung von Kreativität und Partizipation in Gruppenprozessen veröffentlicht: Die „Liberating Structures“ umfassen (aktuell) 33 Prozesselemente, die von Keith McCandless und Henri Lipmanowicz zusammengetragen und kuratiert, teils auch selbst entwickelt wurden (Lipmanowicz und McCandless 2013). Unter den Structures finden sich bekannte Methoden, wie das „World Café“, „Fishbowl“ oder „Open Space“, aber auch völlig neue und teils ungewöhnliche Ansätze. So verwendet z. B. das „Improv Prototyping“ Elemente des Improvisationstheaters, die Technik „TRIZ“ nähert sich der Problemlösung durch destruktive Fragestellungen und „Heard, Seen, Respected (HSR)“ lehnt sich an die „Seed of Compassion“ des Dalai-Lama an.

Sämtliche Structures sind darauf ausgelegt, wirklich alle Teilnehmer*innen einer Gruppe, gleich welcher Größe, aktiv zu beteiligen. Mehrere Structures betten dafür eine „1-2-4-All“ Kommunikationsaufbau ein, in der Teilnehmende zuerst alleine Notizen machen, sich dann zu Zweit austauschen, im Anschluss zu Viert und zum Schluss im Plenum alle Notizen zusammenführen. Für die Notizen werden in Präsenzveranstaltungen Karteikarten auf Pinnwänden verwendet. In online Veranstaltungen gibt es hilfreiche Tools, um eine ähnliche Sichtbarkeit zu erreichen (siehe unten für Details). Mit dieser und weiteren Methoden würdigen die Liberating Structures Meinungsvielfalt, fördern Kreativität und bieten Raum für Selbstreflexion.

### Wo Postdigitale Partizipation erforscht wird: der LWC PdP

Eine seit 2019 geförderte Kooperation zur Erforschung innovativer Formate der Teilhabe und gleichzeitig Praxisbeispiel dieses Beitrages, ist der Leibniz WissenschaftsCampus – Postdigitale Partizipation – Braunschweig (LWC PdP).

Der LWC PdP ist eine interdisziplinäre Forschungspartnerschaft unter Leitung des Georg Eckert Instituts – Leibniz-Institut für internationale Schulbuchforschung mit der Technischen Universität Braunschweig, der Ostfalia Hochschule für angewandte Wissenschaften, dem Deutschen Schifffahrtsmuseum – Leibniz-Institut für Maritime Geschichte, dem Leibniz-Institut für Wissensmedien und dem Haus der Wissenschaft Braunschweig GmbH. Schwerpunkt des LWC PdP ist die gesellschaftliche Teilhabe in einer Welt, in der hybride analog-digitale Technologien und Praktiken unser Leben prägen. Die Ziele, die in Braunschweig verfolgt werden, liegen in der Vermessung und Gestaltung eines neuen interdisziplinären Forschungsfeldes und dem Aufbau internationaler, interdisziplinärer Netzwerke, in der Entwicklung digital gestützter Partizipationsformen mit lokalem und regionalem Fokus sowie in der Gestaltung öffentlicher Diskurse zu (Post‑)Digitalität und Partizipation. Um dies zu erreichen, werden in gemeinsamen Forschungsprojekten sowie einer wissenschaftlichen Nachwuchsforschungsgruppe die Möglichkeiten des Einsatzes hybrider analog-digitaler Technologien und Praktiken zur Förderung von Partizipation in Bildung und städtischem Zusammenleben erforscht, gestaltet und reflektiert. Realer Mittelpunkt des virtuellen WissenschaftsCampus ist „The Basement“, das Digital Lab auf dem Gelände des Georg-Eckert-Instituts. Dieses ist konzipiert als Ort des Austausches für Wissenschaftler*innen aus Kultur‑, Sozial- und Technikwissenschaften mit lokalen Interessenvertreter*innen und wird die regionalen Diskurse der diversen Perspektiven im Themenfeld der postdigitalen Partizipation fördern.

## „Campus Days: Vision“

Die LWC PdP-Jahresveranstaltung „Campus Days: Vision“, die im November 2020 stattfand, wird für den vorliegenden Artikel als Praxisbeispiel für ein postdigitales Konzept herangezogen und nachfolgend näher beschrieben.

### Veranstaltungsziele und Programmablauf

Nachdem im November 2019 der WissenschaftsCampus offiziell eröffnet wurde und die organisatorische Basis für den LWC PdP vorbereitet war, sollte im Herbst 2020 ein wissenschaftlicher Austausch unter allen ca. 50 Mitgliedern und Assoziierten des Campus zu spezifischen Begrifflichkeiten, Ansätzen und Methoden stattfinden. Da sich seit der Antragsphase die personelle Zusammensetzung der Beteiligten verändert hatte und neu hinzugekommene Promovend*innen, Projektleiter*innen und Beiräte einander noch nicht bekannt waren, sollten auch Vernetzungsformate in die Veranstaltung integriert werden. Desweiteren galt es, die 2018 im Antrag formulierten Ziele und Inhalte auf Aktualität zu überprüfen und eine gemeinsame Vision für die Zukunft des Campus zu kreieren. Zusammenfassend können folgende **Ziele für die Veranstaltung** festgehalten werden:

- A. Erarbeitung einer gemeinsamen Vision für die Zukunft des LWC PdP
- B. Annäherung an die Vision: Identifikation von Hindernissen, Gestaltung der Zusammenarbeit und Einbezug weiterer Stakeholder
- C. Gestaltung der Arbeitsebene, Verständigung über Strukturen und Prozesse
- D. Planung der nächsten Aktivitäten
- E. Vorstellung von Projekten und Beteiligten des LWC PdP
- F. Kennenlernen der Beteiligten, Austausch und Vernetzung

Diese Ziele sollten in einem Tagesprogramm mit Zeitrahmen von 9:00 Uhr bis 16:00 Uhr erreicht werden. Dabei teilte sich die Veranstaltung in zwei Teile: Während des Vormittags (9:00 Uhr bis 13:00 Uhr) waren die Mitglieder der Beiräte (Nutzer*innenbeirat und wissenschaftlicher Beirat) eingeladen mitzuwirken. Hier sollten speziell die Ziele A, B, E und F verwirklicht werden. Nach Verabschiedung der Beiräte am Mittag, fokussierte die Veranstaltung die Ziele C und D. Nachfolgend ist der Ablauf tabellarisch dargestellt, wobei die Ziele A–F den TOPs zugeordnet sind (Tab. 1):

**Tab. 1:** Tagesablauf und Zielverwirklichung der LWC PdP-Veransaltung

Tagesordnungspunkte (TOPs)		Ziele
01	WELCOME: Begrüßung, Agenda und Technik	–
02	PROJECTS: Vorstellung der LWC PdP-Projekte	E, F
03	VISIONS: Erarbeitung einer Vision	A, F
04	PRINCIPLES: Annäherung an die Vision	B, F
05	PARTICIPANTS: Austausch über Stakeholder	B, F
06	STRUCTURES: Gestaltung der Arbeitsebene	C, F
07	PRACTICES: Arbeitsplan und Aktivitäten	D, F
08	FINAL ROUND: Zusammenfassung	D

### Technische und organisatorische Rahmenbedingungen

Die Organisation und Durchführung der Veranstaltung erfolgte in einem Team mit getrennten Verantwortlichkeiten. Die inhaltliche Gestaltung übernahm die Campus-Leitung, die technische Verantwortung ein Konsortialpartner mit Expertise im wissenschaftlichen Eventmanagement. Eine externe Moderatorin führte durch den Tag, wobei die Moderation in Bewegung erfolgte und dem physischen Setting besondere Bedeutung zukam: Beleuchtung (Studioleuchten-Set), Ton (tragbarer Sprachverstärker, Mischpult, Lautsprecher), Übertragung (externe Bildschirme, LAN-Verbindung) und Raum (Platz, Hintergrund, erhöhte Ablage) wurden bewusst arrangiert und getestet.

Die technische Basis der Veranstaltung bildete „Zoom“, ein Enterprise-Videokonferenzsystem mit Instant-Messaging und Content-Sharing, das die Einrichtung von bis zu 50 Gruppenräumen (Break-Out-Räumen) erlaubt und keine Anmeldung zur Teilnahme erfordert. Der Veranstaltungslink wurden den Teilnehmer*innen der LWC PdP-Veranstaltung im Vorfeld übermittelt, zusammen mit Terminen für technische Proben für die mit dem Videokonferenzsystem weniger versierten Teilnehmer*innen. Um einen reibungslosen Ablauf zu erreichen, Störungen zu vermeiden und die System-Performance stabil zu halten, wurden im Plenum Kameras und Mikrofone von Teilnehmer*innen ohne Redebeitrag stets ausgeschaltet; Fragen konnten im Zoom-Chat platziert werden.

Die Nutzung des Tools „Miro“ ergänzte den technischen Rahmen. Hierbei handelt es sich um ein online-Whiteboard, das die gleichzeitige Zusammenarbeit mehrerer Personen auf einer potenziell endlos skalierbaren Leinwand (Canvas) ermöglicht. Grafische Elemente, Textfelder, Bilder und Freihandzeichnungen sind ebenso integrierbar wie Ansichtsfenster für diverse Dokumente (Word, PPT, PDFs) und Abspielfenster für interaktiven Content (YouTube-Videos, Google Maps-Daten). Einzelne Objekte können von Nutzer*innen mit Kommentaren versehen und verschlagwortet werden. Zur Übersicht lässt sich das Canvas eines Boards in Themenbereiche („Frames“) strukturieren. Ein freigegebenes Board ist per Link ohne Anmeldung nutzbar.

### Formate und eingesetzte Methoden im Tagesablauf

#### Top 01) Welcome

Rund 30 min vor Beginn stand der Zoom-Veranstaltungsraum offen, sodass etwaige Schwierigkeiten von Teilnehmer*innen mit Audio- und Videoübertragung durch den technischen Support geklärt werden konnten. Die Begrüßung inkl. Einführung in den Tagesablauf wurde mit einer über die Content-Sharing-Funktion gezeigten PowerPoint-Präsentation durchgeführt. Im Anschluss wurde das für die Veranstaltung eingerichtete Miro-Board live geöffnet. Der Link hierzu wurde in den Zoom-Chat gestellt. Die Moderatorin gab eine Einführung in die wichtigsten Miro-Funktionen und in die für die Veranstaltung vorbereitete Struktur.

#### Top 02) Projects

**Abb. 1:**
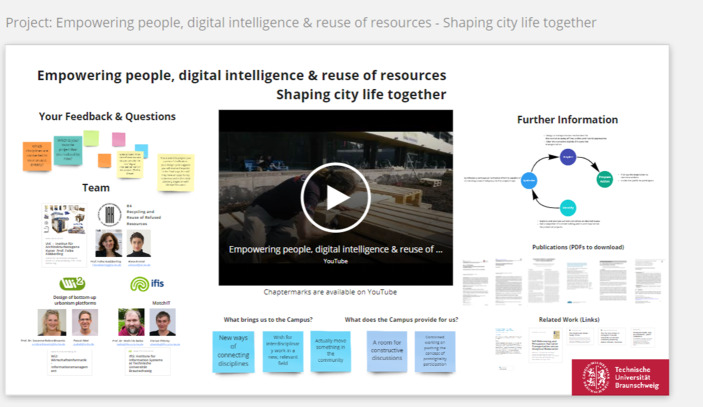
Eine der Projektpräsentationen im Miro-Board der Veranstaltung des WissenschaftsCampus – Postdigitale Partizipation – Braunschweig

Die Veranstaltung wurde mit einer halbstündigen Projektausstellung eröffnet. Hierfür hatte jedes Projektteam im Vorfeld eine digitale Präsentation in Miro erstellt. Ähnlich wie bei einer Ausstellung im analogen Raum, stand jedem Projekt eine Fläche (ein eigener Frame) zur Verfügung. Diese wurde mit multimedialen Inhalten, Informationen zu den Projektbeteiligten, Informationsmaterial, Kontaktdaten und einem Feedback-Bereich gefüllt (vgl. Abb. 1) und konnte zeitgleich von allen Teilnehmer*innen der Veranstaltung erkundet werden.

#### Top 03) Visions

**Abb. 2:**
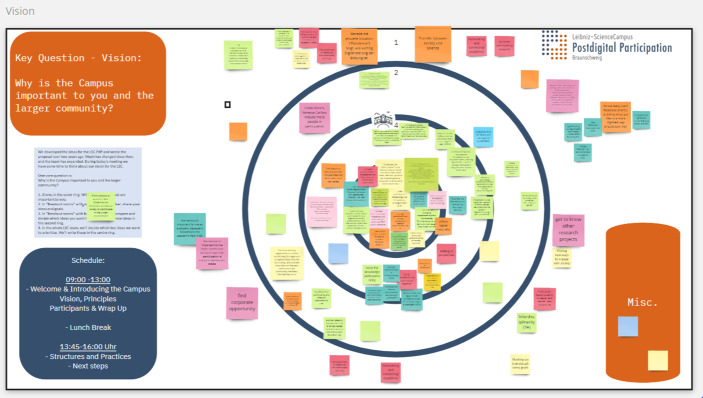
Ergebnisse der 1‑2-4-All Structure im Miro-Board; aus Platzgründen hier nur zur Veranschaulichung des Designs, nicht zum Lesen der Inhalte

Im Zoom-Plenum wurde den Teilnehmer*innen das weitere Vorgehen zur gemeinsamen Zusammenarbeit erläutert. Zunächst wurde die im weiteren Ablauf relevante 1‑2-4-All-Methode (vgl. Abschnitt 2.2) vorgestellt, dann auf das Prinzip der Breakout-Räume eingegangen. Im Anschluss wechselten alle Teilnehmer*innen in das Miro-Board zum vorbereiteten Frame „Vision“. Im Zentrum des Frames waren drei große Kreise platziert (vgl. Abb. 2), am Rande die zu bearbeitende Fragestellung und einige Hilfetexte. Zur Bearbeitung der ersten Kernfrage („Warum ist der Campus für dich persönlich und für die Gesellschaft von Bedeutung?“) erhielten die Teilnehmer*innen 2 min Zeit. Eigene Gedanken konnten über virtuelle Notizzettel außerhalb des äußersten Kreises abgelegt werden. Nach Ablauf des Timers erfolgte eine zufällige Einteilung von zwei Personen in einen Breakout-Raum. Für den bilateralen Austausch standen 8 min zur Verfügung. Die gemeinsamen Antworten wurden erneut in Notizzetteln festgehalten, die im äußersten Innenkreis angeordnet wurden. Im nächsten Zyklus wurden je zwei Breakout-Räume zu 4er-Gruppen zusammengefügt, die 10 min Bearbeitungszeit im nächstinneren Kreis erhielten. Im Anschluss gelangten die Teilnehmer*innen zurück ins Plenum, wo die Moderatorin in einer 30-minütigen Abschlussrunde eine Diskussions-Zusammenfassung der 4er-Gruppen erfragte. Die als relevantesten eingestuften Kernaussagen wurden im Zentrum der Kreise zusammengeführt, wie Abb. 2 im Ergebnis zeigt.

#### Top 04) Principles

Mit der nun geläufigen Arbeitsweise und Methode, wechselten alle Teilnehmer*innen in den Frame „Principles“, zur zweiten Kernfrage („Wie gelingt es uns, unsere Vision zu erreichen?“). Hier sollten gemeinsame Grundsätze erarbeitet werden, die zur Erreichung der Vision beitragen. Analog zum oben beschriebenen Vorgehen waren drei Bearbeitungsrunden in Breakout-Räumen angesetzt. Die Beiträge wurden sukzessiv von außen nach innen übertragen, bis hin zur Konsolidierung im Zentrum.

#### Top 05) Participants

In der nächsten Arbeitsrunde wurde in einem analog zu VISION und PRINCIPLES vorbereiteten Frame die dritte Kernfrage („Wer muss noch in den Campus eingebunden werden, damit wir unsere Vision erreichen?“) bearbeitet. Der Ablauf und die zeitlichen Vorgaben blieben gleich, jedoch entfiel nach der Diskussion in 4er-Gruppen die Konsolidierung im Plenum. Stattdessen wurde im Frame „Wrap Up“ eine zwischenzeitlich erstellte Zusammenfassung der Ergebnisse des Vormittags präsentiert. Der TOP schloss mit Fragen an die Beiräte und ihrer Verabschiedung.

#### Top 06) Structures

Nach der Mittagspause wurde mit dem bisherigen Setting eine weitere Diskussionsrunde durchgeführt, wobei die vierte Kernfrage in Anlehnung an das TRIZ Structure umgedreht formuliert war („Welche Arbeitsweisen sollten wir einführen oder fortführen, um unsere Vision *unerreichbar* zu machen?“). Im 1‑2-4-All-Prinzip wurde über sich negativ auswirkende, hemmende und destruktive Arbeitsstrukturen und -prozesse diskutiert, die Diskussionsergebnisse aus den Gruppen miteinander abgeglichen und Kernaussagen im Frame „Structures“ konsolidiert.

#### Top 07) Practices

Für den vorletzten Programmpunkt stand ein Frame zur Verfügung, dessen Inhalt über zwei rechteckige Rahmen in zwei Gruppen unterteilt war: die Gruppe der Promovend*innen und die der Professor*innen bzw. Projektleiter*innen. Der Meeting-Host teilte die noch Anwesenden entsprechend in zwei Gruppenräume auf, wo sie innerhalb von 20 min die fünfte Kernfrage („Was werden wir als Campus als nächstes angehen, um unserer Vision näher zu kommen?“) diskutierten und dem Plenum kurz vorstellten.

#### Top 08) Final Round

In der Abschlussrunde im Frame „Next Steps“ gaben die Campus-Sprecher*innen eine Übersicht zu den geplanten nächsten Schritten und bevorstehenden Aktivitäten. Die Moderatorin trug offene Fragen und Anregungen zusammen. In einem „Closing Circle“ wurde eine Blitzlicht-Runde visualisiert. Über ein selbstgewähltes und dort eingefügtes Bild, Icon oder Symbol gaben die Teilnehmer*innen ein kurzes Feedback zum Veranstaltungstag.

## Im Zeitgeist von Netzwerken und Communities: die PIP-Formel

Die Covid-19-Pandemie hat den Alltag in allen Bereichen unserer Gesellschaft massiv beeinflusst. Dort, wo flexible Arbeitsmodelle umsetzbar sind, hat sich die Anwesenheit von Mitarbeiter*innen ins Homeoffice verlagert und die flächendeckende Etablierung von Technologien zur Unterstützung von Arbeitsprozessen gefördert. Die Umsetzung von online-Besprechungen hat gezeigt, dass ein Vor-Ort-Sein für die meisten Routine-Termine nicht mehr notwendig ist. Gleichzeitig musste festgestellt werden, dass die unreflektierte Digitalisierung von Workshops und Veranstaltungen kein adäquater Ersatz für selbige sein kann. Etablierte Arbeitsmethoden, die bereits in Präsenz keine kollaborative Herangehensweise ermöglichen, erschweren es im digitalen Raum, die Aufmerksamkeit und das Engagement von allen Beteiligten zu gewinnen. Es ist kaum denkbar, dass dies eine vorübergehende Herausforderung ist und es nach der Corona-Krise von der neuen, flexiblen Arbeitsweise und der zunehmenden Gewöhnung an den Umgang mit digitalen Werkzeugen eine komplette Rückkehr in die frühere Arbeitswelt geben wird. Vielmehr entspricht die aktuelle Entwicklung dem neuen Zeitgeist der Zusammenarbeit.

Aus den Erfahrungen der vergangenen Monate und insbesondere aus denen des in Abschnitt 3 beschriebenen Praxisbeispiels, schlagen wir für die Umsetzung künftiger Veranstaltungsformate die Berücksichtigung folgender Grundsätze vor:

### Postdigital

Digitale Technologien werden weiterhin genutzt, aber die Teilnehmenden verstehen die Settings als *postdigital* – statt nur digital. Mit dem Begriff „postdigital“ zu denken, bedeutet drei Aspekte bei online-Veranstaltungen hervorzuheben.

- Erstens, *die materielle Umgebung* spielt eine subtile jedoch starke Rolle beim Erfolg des online Events. So wirkt z. B. der Klang der Stimme mit einem Headset anders (für viele Zuhörer*innen: „besser“) als ohne.
- Zweitens, *der Körper* spielt mit. Redner*innen, die im Stehen präsentieren, können ihre Körpersprache zum Einsatz bringen. Zunehmend finden wir, auch zuhause, eine Tischerhöhung, um uns gelegentlich im Stehen am Gespräch zu beteiligen.
- Drittens, *die Sozialität* ist für das Gelingen essentiell. Bei face-to-face Veranstaltungen wissen alle Organisator*innen, wie wichtig Kaffeepausen sind. Für Videokonferenzen werden kreative Lösungen entwickelt, um Zweier-Gespräche und Gesprächen in kleinen Gruppen zu ermöglichen. Diese müssen in online-Veranstaltungen noch expliziter in den Ablauf eingebunden sein.

Dieser Beitrag beschrieb die 1‑2-4-All Methode in Breakout-Räumen als einen erfolgsversprechenden Ansatz, um online ähnlich energetisierende, motivierende und beteiligungsverstärkende Effekte zu erzielen, wie sie soziale Pausen in Präsenz bieten. Aber nicht nur online-Veranstaltungen sind postdigital: auch andersherum gilt es, Präsenzveranstaltungen postdigital zu denken. Wie, zum Beispiel, ermöglichen wir in Präsenz die kommentierende Diskussion nebenbei, die im Chat für alle Teilnehmenden sichtbar ist?

### Interaktiv

Passiv ist passé. Weder in analogen, digitalen oder postdigitalen Veranstaltungen möchten die Teilnehmenden nur passive Zuhörer*innen sein. Noch sollte es im Interesse der Organisator*innen liegen, ihnen diese Rolle zuzuordnen. Jede Interaktion ist wertvoll und bietet die Chance, eine authentische Verbindung zu einer Person aufzubauen, z. B. für Feedback, Aufmerksamkeit, Netzwerken oder Kooperationen. Für Veranstaltungen im rein digitalen Raum gilt noch mehr als in Präsenz, die Aufmerksamkeit der Teilnehmer*innen zu halten. Sowohl im Netz als auch im Homeoffice warten zahlreiche Ablenkungen und erschweren es, den Inhalten zu folgen. Die Einbindung der Zuhörer*innen *vorher, währenddessen* und *danach*, schafft Verbindung und erhöht auf beiden Seiten den Mehrwert der Veranstaltung. Es reicht aber nicht aus, nur in Interaktionen zwischen Redner*in und Zuhörerschaft zu denken, wie sie durch Votings, direkte Fragen, Social Walls, Feedbackrunden oder Applaus im analogen oder digitalen Raum umgesetzt werden können. Es gilt vor allem auch, den Austausch zwischen den Teilnehmenden zu ermöglichen. Der Networking-Faktor, das Entstehen von Kontakten und das Kennenlernen neuer Personen sollten auf keiner Veranstaltung fehlen.

### Partizipativ

Partizipative Führung, partizipative Veranstaltungen, partizipative Gestaltungsprozesse usw. sind zukünftig mehr als ein Lippenbekenntnis. Partizipation ist zum eindeutig positiv konnotierten Wort geworden. Aber, wie bei vielen solchen Prinzipien bestehen diverse Operationalisierungen, die die Partizipation aller Teilnehmenden mehr oder weniger ermöglicht. Ziel sollte sein, die Ergebnisse einer Veranstaltung – ob es um Entscheidungen, Strategien, Gedankenaustausch, Feedback oder Visionen geht – von allen Teilnehmenden mitentwickeln zu lassen. Expertise und Erfahrungen aus verschiedenen Statusgruppen, Hierarchieebenen und Lebensbereichen werden mitgeteilt und finden Gehör. Dazu sind diverse Beteiligungsformate notwendig. Die Diversität ist am sichtbarsten in der Verwendung von schriftlichen Stickies in online-Whiteboardtools wie Miro oder Mural sowie in Liberating Structures wie „1-2-4-All“. Sowohl die schriftliche Mitteilung erster Ideen als auch das Gespräch im geschützten Raum einer Kleingruppe ermöglichen es, ruhigere oder introvertierte Personen in einem frühen Stadium zu beteiligen, ohne den Druck des öffentlichen Redens. Diese Ideen werden in der Regel aufgegriffen und von anderen, die sich im Plenum wohler fühlen, weitergetragen. Ergebnisse, die in dieser Form von möglichst vielen Beteiligten mitgestaltet werden, werden von vielen als gemeinschaftlich erarbeitetes Ergebnis – aus gemeinsamer Kraft statt top-down – mitgetragen.

## Gemeinsam in die Post-Corona-Zeit

Basierend auf dem Ergebnis der „Campus Days: Visions“ sehen wir in der Anwendung der PIP-Formel (Postdigital, Interaktiv, Partizipativ) auch für zukünftige Veranstaltungen in der Post-Corona-Zeit ein großes Potenzial. Mit der Wahl der Methoden ist es gelungen, die Ziele der Veranstaltung umzusetzen und gleichzeitig eine positive Resonanz zu erzielen. Dazu beigetragen haben auch der technisch reibungslose Ablauf und die Gesamtmoderation. So wurde z. B. die gründliche Einführung in Methoden und Tools auch von Teilnehmer*innen mit Vorwissen als sehr hilfreich empfunden. Während des Tagesablaufs und in der Abschlussrunde haben uns die Teilnehmer*innen wertvolles Feedback gegeben. Rückmeldungen, wie z. B. „Ich hatte zunächst Bedenken vor den sieben Stunden online-Veranstaltung, aber es hat viel Spaß gemacht“, „Für mich die beste Videokonferenz des Jahres“ oder „Ich habe sogar neue Leute kennengelernt.“ sind natürlich Bewertungen, die Organisator*innen gerne hören. Da zum Zeitpunkt der Durchführung jedoch alle Teilnehmer*innen bereits mehrere Monate im Homeoffice waren, kann von Erfahrungswerten mit online-Veranstaltungskonzepten ausgegangen werden. Vor dem Hintergrund der aufgezeigten gesellschaftlichen Entwicklungen sind wir davon überzeugt, dass nur ein Weg erfolgreich durch die Postdigitalität führt. Es ist der gleiche, den wir durch die Pandemie nehmen: GEMEINSAM.
